# Factors Influencing Deoxynivalenol Accumulation in Small Grain Cereals 

**DOI:** 10.3390/toxins4111157

**Published:** 2012-11-06

**Authors:** Stephen N. Wegulo

**Affiliations:** Department of Plant Pathology, University of Nebraska-Lincoln, Lincoln, NE 68583, USA; Email: swegulo2@unl.edu; Tel.: +1-402-472-8735

**Keywords:** Fusarium head blight, deoxynivalenol, small grain cereals, environmental factors, growth stage, inoculum dosage, pathogen aggressiveness, chemotype, lodging, tillage system, cultivar resistance, fungicide application, regulatory/advisory standards

## Abstract

Deoxynivalenol (DON) is a mycotoxin produced by the plant pathogenic fungi *Fusarium graminearum * and *F. culmorum*. These and other closely related fungi cause a disease known as Fusarium head blight (FHB) in small grain cereals. Other mycotoxins produced by FHB-causing fungi include nivalenol, T-2 toxin, and zearalenone. Ingestion of mycotoxin-contaminated food and feed can lead to toxicosis in humans and animals, respectively. DON is the predominant and most economically important of these mycotoxins in the majority of small grain-producing regions of the world. This review examines the factors that influence DON accumulation in small grain cereals from an agricultural perspective. The occurrence and economic importance of FHB and DON in small grain cereals, epidemiological factors and cereal production practices that favor FHB development and DON accumulation in grain under field conditions, and regulatory/advisory standards for DON in food and feed are discussed. This information can be used to develop strategies that reduce DON accumulation in grain before harvest and to mitigate the human and animal health risks associated with DON contamination of food and feed.

## 1. Introduction

Deoxynivalenol (DON), also known as vomitoxin, is a trichothecene mycotoxin produced by the fungal plant pathogens *Fusarium graminearum* (sexual stage: *Gibberella zeae*) and *F. culmorum * [1,2]. Both pathogens and several other species of *Fusarium* and its allies cause a disease known as Fusarium head blight (FHB) in wheat, barley, and other small grain cereals [1,2,3,4]. *F. graminearum* also causes ear and stalk rots in maize [5]. In addition to DON, the trichothecenes nivalenol (NIV) and T-2 toxin and the sterol zearalenone (ZEA) are produced by FHB-causing pathogens [6,7]. These mycotoxins are harmful to humans and animals. Although DON is the least toxic of them, it is the most commonly detected *Fusarium* mycotoxin [8] and is the predominant and most economically important mycotoxin in small grain production [1]. Therefore, this review examines the factors that influence the accumulation of DON in small grain cereals under field conditions. An understanding of these factors can be useful in devising strategies aimed at reducing the amounts of DON that accumulate in grain before harvest and, ultimately, in mitigating the human and animal health risks associated with DON contamination of food and feed. 

**Figure 1:**
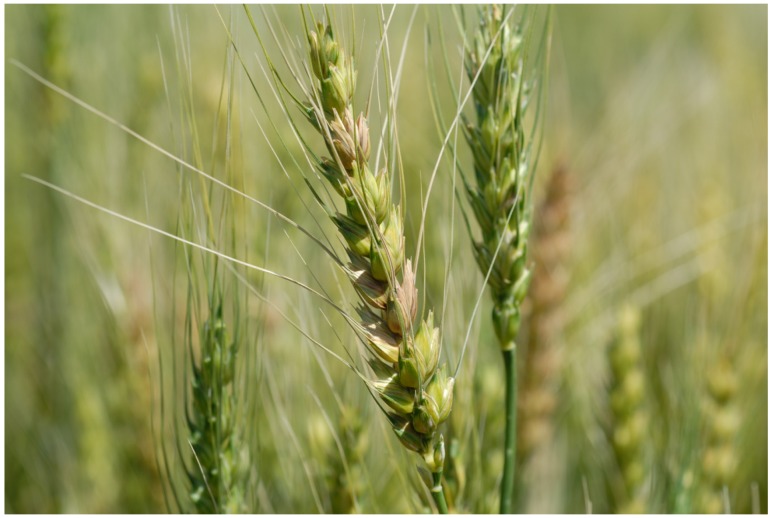
A wheat spike with a few spikelets bleached following natural infection by *Fusarium graminearum* in the field.

**Figure 2:**
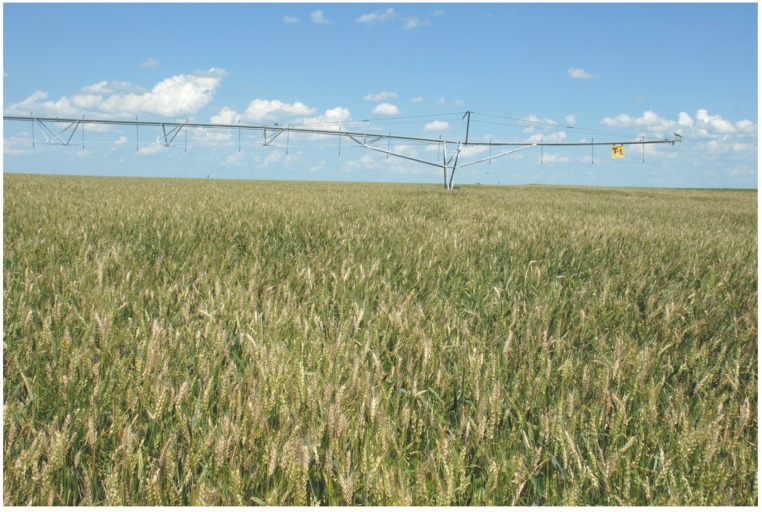
A wheat field affected by a severe Fusarium head blight (FHB) epidemic in south central Nebraska, USA in 2008.

**Figure 3:**
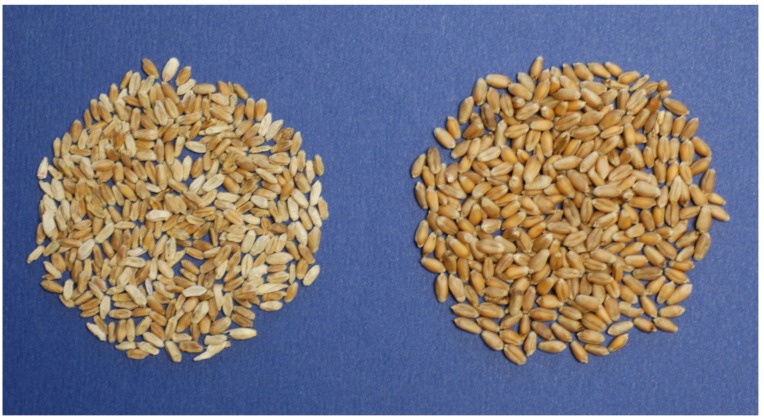
Wheat grain damaged by *Fusarium* (left) and healthy grain.

During the growing season in the field, FHB causes premature bleaching of spikes of small grain cereals. In wheat, the bleaching starts with one or more spikelets on a spike (Figure 1) and can continue until the entire spike is whitened. In a disease-favorable growing season, numerous spikes can be seen randomly scattered in the field (Figure 2). Bleached spikelets are sterile or contain shriveled and/or chalky white or pink kernels known as *Fusarium*-damaged kernels (FDK), scabby kernels, or “tombstones” [9] (Figure 3). Kernels that appear to be healthy also may be infected, especially if infection occurred late in kernel development. Infected kernels contain mycotoxins, primarily DON. In controlled experiments, DON was detected as early as 36 hours after inoculation of wheat spikelets with *F. culmorum* [10]. 

**Figure 4:**
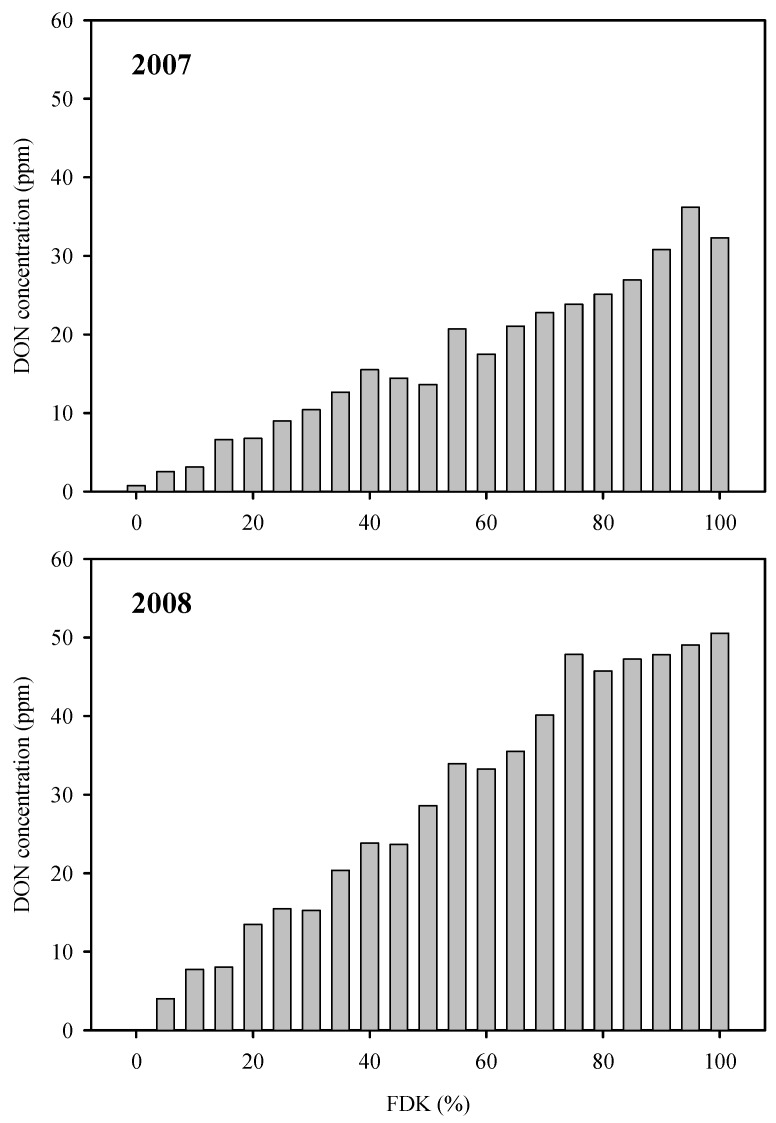
Deoxynivalenol (DON) concentration in wheat grain samples with an increasing proportion (by weight) of *Fusarium*-damaged kernels.

DON concentration in *Fusarium*-damaged grain generally increases with the percentage of damaged grain in a given sample. To demonstrate this, the author mixed wheat *Fusarium*-damaged kernels (FDK) with healthy kernels in 5% (by weight) increments from 0% FDK, 100% healthy kernels to 100% FDK, 0% healthy kernels. The FDK were obtained by separating them from grain collected from winter wheat fields and grain elevators in 2007 and 2008 when there were severe FHB epidemics in Nebraska, USA. Subsamples of grain from each mixture were ground to flour and submitted to the North Dakota Veterinary Diagnostic Laboratory for DON content determination using gas chromatography with electron capture detection (GC/ECD) [11]. In both years, DON concentration increased with the percentage of FDK in grain samples (Figure 4).

The acetylated derivatives of DON, 3-ADON and 15-ADON, are commonly detected in contaminated grain [12]. Miller *et al.* [13] showed that DON-producing isolates of *F. graminearum* collected from different regions of the world differed in their production of 3-ADON and 15-ADON. Some isolates produced one or the other type of acetylated derivative, whereas others produced both types. For those isolates that produced either 3-ADON or 15-ADON but not both, there was a correlation between geographical origin and isolate chemotype. Isolates from China produced predominantly 3-ADON whereas isolates from Mexico and North America produced mainly 15-ADON.

In humans, food poisoning characterized by diarrhea, nausea, vomiting, abdominal pain, headache, dizziness, and fever has been associated with consumption of *Fusarium*-infested cereals or food products [1,14,15]. Desjardins [1] discussed some historical case studies of *Fusarium* mycotoxin-associated illnesses. They include human alimentary toxic aleukia in Russia and Central Asia, described in 1932 by Russian scientists. The disease was characterized by the food poisoning symptoms described above in mild cases and often fatal skin rashes and necrotic lesions of the alimentary tract and other organs in serious cases. It was associated with consumption of wheat, barley, and other cereal grains left in the field during the winter and harvested in the spring. At about the same time, Japanese scientists linked outbreaks of a human toxicosis to the consumption of cereal grains, including wheat and barley, discolored by a red mold disease known as akakabi-byo. In the United States, swine feed refusal accompanied by vomiting, weakness and emaciation occurred when the animals were fed wheat, barley and oat grain harvested from fields that were affected by a severe FHB epidemic in the central states in 1928. *F. graminearum* was the predominant *Fusarium* species isolated from the grain. 

Trichothecenes have multiple effects on eukaryotic cell functions; the primary effect is believed to be inhibition of protein synthesis [16]. Although DON is the least toxic of the trichothecene mycotoxins produced by *Fusarium* spp. in small grains, it can cause significant harm to humans and animals when ingested in large quantities. DON has been shown to inhibit the absorption of certain nutrients by human intestinal epithelial cells [17]. In animals, clinical signs of trichothecene toxicosis include feed refusal and weight loss, emesis, hemorrhage, and cellular necrosis of mitotically active tissues such as the intestinal mucosa, skin, and bone marrow [16]. 

In plants, DON and 3-ADON have been shown to be phytotoxic. Wang and Miller [18] showed that of several *F. graminearum* metabolites, DON and 3-ADON most strongly inhibited growth of wheat coleoptile tissue. Growth of the coleoptile tissue of most of the 14 spring wheat cultivars tested was inhibited at a DON and 3-ADON concentration of 10^−6^ M, and inhibition was much stronger at higher concentrations. Bruins *et al.* [19] exposed seedlings, coleoptile segments, anther-derived callus, and anther-derived embryos to DON and 3-ADON. They found that DON inhibited growth of all four types of plant material. Shimada and Otani [20] found DON to strongly inhibit root growth in seedlings of seven wheat cultivars and suggested, based on their observation, that DON might be useful in selecting cells resistant to *F. graminearum* in cell cultures. Because DON is water soluble [21], it can be translocated to other parts of the plant where it can exert physiological effects. Kang and Buchenauer [10] found DON and 3-ADON in mycelium-free wheat plant tissues distant from *F. culmorum*-inoculated spikelets. They concluded that the toxins can be translocated upwards via xylem vessels and phloem sieve tubes, and downward via phloem sieve tubes.

## 2. Occurrence and Economic Importance of FHB and DON in Small Grain Cereals

FHB occurs worldwide and especially in temperate regions where small grain cereals (wheat, barley, triticale, oats, rye) are grown [22,23]. It is caused by several species of *Fusarium* and its allies, notably *F. graminearum, F. culmorum, F. avenaceum, F. poae, * and *Microdochium nivale* [4,23]. *F. graminearum* and *F. culmorum* are the most pathogenic and most common [22]. These fungi produce mycotoxins, mainly trichothecenes, which contaminate grain. Their geographical distribution appears to be influenced by climate. For example, *F. graminearum* most commonly occurs in North America, Southern China, and Eastern Europe whereas *F. culmorum* is common in cooler areas such as Western Europe [2,13,22,24].

FHB can cause significant economic losses. Johnson *et al.* [25] estimated that FHB caused direct losses in wheat and barley totaling more than $1.3 billion in the U.S. during the period from 1991 to 1997. They estimated the total economic impact in rural communities and businesses related to grain production and marketing to be three to four times this amount. Direct losses are manifested as reduced yield due to FDK or spikelet sterility; low test weight of harvested grain; grain cleaning costs to remove FDK; and price discounts at grain elevators due to DON contamination. In addition, germination of *Fusarium*-infected grain can be reduced to unacceptable levels [26] and if the grain is used as seed for the next season’s crop, seedling blights and root and crown rots can significantly reduce stand establishment and subsequently yield. In years in which severe and widespread FHB epidemics occur, there can be scarcity of seed needed to plant the next season’s crop. Indirect losses result from poor quality food products (e.g., bread and beer) made from *Fusarium*-damaged and DON-contaminated grain; reduced productivity of livestock due to toxicosis or feed refusal; costs incurred in the treatment of toxicosis in humans and animals; costs associated with fungicide seed treatment to control seedling and root and crown rot diseases; and loss of profits or failure of businesses related to grain production and marketing. Worldwide, losses due to FHB can be staggering in years when environmental conditions favor disease development.

## 3. FHB Disease Cycle

Because some of the factors that influence the accumulation of DON in small grain cereals include or are related to pathogen growth stages, a brief description of the disease cycle of FHB is provided. *F. graminearum* overwinters as chlamydospores or mycelia in the soil or in host crop residues which serve as a source of primary (initial) inoculum in the spring [3]. The fungus can also survive on wheat seed. Primary inoculum mainly consists of ascospores produced in perithecia (sexual fruiting structures), which form on crop residues in the spring as temperatures warm up. In the spring, ascospores and/or conidia are released from crop residues and spread by wind and/or splashing water from rain or irrigation. They land on spikes and during wet, warm weather they germinate and infect glumes, flower parts, or other parts of the spike. Infections occur mostly during anthesis (stage at which anthers rupture and shed pollen during flowering) partly because the anthers contain stimulants for spore germination and pathogen growth [27]. Spikes are susceptible from anthesis until the soft dough stage. Infections that occur during anthesis are the most damaging. During warm temperatures (25 °C to 30 °C) and wet conditions, blight symptoms develop within 2 to 4 days after infection. Therefore, an apparently healthy crop can show symptoms suddenly. Later in the growing season or after harvest, perithecia may form on spikes. FHB is considered a monocyclic or one cycle disease, that is, after the initial or primary infection, little or no secondary infection occurs by conidia formed on infected spikes.

## 4. Factors Influencing DON Accumulation in Grain

The amount of DON produced by *F. graminearum* is positively correlated with fungal biomass. Using real-time polymerase chain reaction (PCR) analysis, Demeke *et al.* [28] quantified *F. graminearum* DNA in grain of two classes of Canadian wheat (western red spring and western red winter). They also measured DON in the same grain using gas chromatography with mass selective detection. Linear regression of DON on fungal DNA revealed a strong, positive relationship (*R*^2^ ≥ 0.90) between the two variables in both classes of wheat. Sneller *et al.* [29] similarly showed that in *F. graminearum*-susceptible wheat genotypes, fungal biomass had a strong, positive correlation with DON. Therefore, when discussing the factors that affect DON accumulation in grain, it is assumed that factors that promote pathogen growth in a susceptible host also promote DON production. Pathogen growth and expression of pathogenicity in the host are quantified indirectly as disease intensity (FHB incidence, severity, or index). 

**Figure 5:**
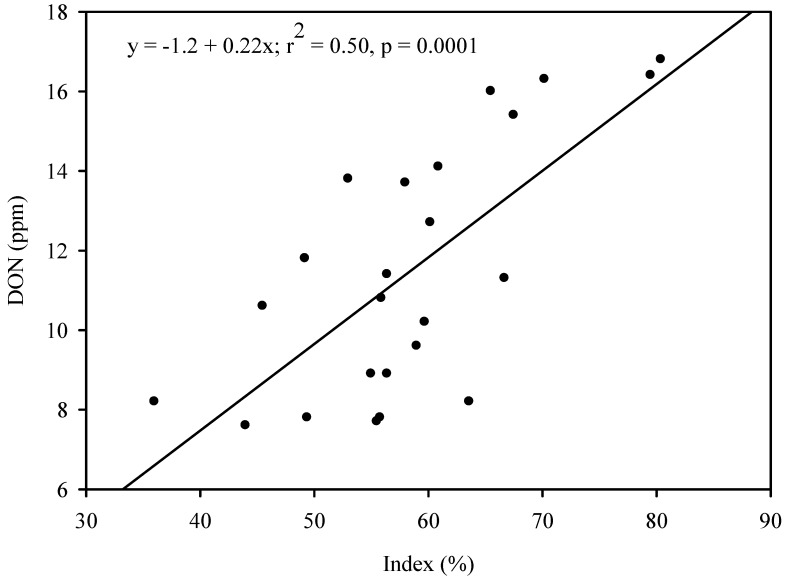
Relationship between FHB index and DON concentration in grain from a field experiment in which different levels of FHB intensity were generated by applying or not applying the fungicide Prosaro to two winter wheat cultivars.

Field studies have demonstrated a positive, linear relationship between FHB intensity and DON [30,31,32]. In 2008, when there were severe FHB epidemics in wheat fields in Nebraska and other states in the FHB-prone regions of the U.S., the author generated, in a field experiment, different levels of FHB intensity (measured as index (%) = [incidence (%) × severity (%)]/100) by applying or not applying the fungicide Prosaro (prothioconazole + tebuconazole) to two FHB-susceptible winter wheat wheat cultivars, ‘2137’ and ‘Jagalene’ at early anthesis. All plots were spray-inoculated with *F. graminearum* spores (1 × 10^5^ spores/mL) 24 hours after fungicide application. In addition, maize kernels colonized by *F. graminearum* had been spread on the soil surface (0.42 g/m^2^) in the plots one week before anthesis. Following rehydration of the maize kernels by natural rain, mycelial growth of the fungus culminated in sporulation, which provided additional spore inoculum during anthesis. The experimental design was a split plot in randomized complete blocks with six replications. The main plot and subplot treatments were cultivar and fungicide application (Prosaro or no Prosaro), repectively. Index was measured 21 days after spray-inoculation. Following harvest, a grain subsample from each plot was ground to flour and submitted to the North Dakota Veterinary Diagnostic Laboratory for DON content determination using gas chromatography with electron capture detection (GC/ECD) [11]. Regression of DON concentration on index using plot data revealed a significant, positive linear relationship between the two variables (Figure 5). Similar results were obtained from replicated field studies using the same methodology conducted over three years in Kansas and Nebraska, USA [30].

Other field studies have similarly shown a positive, linear relationship between DON concentration and FHB intensity. Hernandez Nopsa *et al.* [32] tagged spikes of two winter wheat cultivars in increasing FHB severity categories and, following harvest, submitted ground subsamples of grain for DON content determination as described above. They found significant, positive, linear relationships (correlation coefficients ranging from 0.57 to 0.77) between FHB severity and DON concentration in both cultivars in all the three years during which the study was conducted. Paul *et al.* [31] used meta-analysis to analyze 163 published and unpublished field studies. They found more than 65% of correlation coefficients between measures of FHB intensity (incidence, severity, index) to be >0.50. The analysis determined that the relationship between diseased spike severity and DON concentration was linear and positive with a mean correlation coefficient of 0.53. The results from these studies indicate that FHB intensity is a major factor influencing DON accumulation in small grain cereals. Therefore, factors that influence FHB development will also influence DON accumulation in grain. 

### 4.1. Environmental Factors

Temperature, moisture, and relative humidity (RH) are the most important environmental factors influencing the development of FHB and therefore DON accumulation in small grain cereals. Climate change can greatly influence these factors by causing excessive rainfall or drought [33]. Andersen [34] conducted detailed experiments to determine the effects of temperature and moisture on FHB development in spring wheat. Plants were inoculated with conidia of *F. graminearum* at the past flowering growth stage (kernels nearly filled and in the milk to soft dough stage) and incubated at temperatures ranging from 15 to 30 °C with exposure to continuous wetness for periods ranging from 24 to 60 hours. Results showed that the optimum temperature for FHB development was 25 °C regardless of the duration of continuous wetness. Longer periods of exposure to continuous wetness resulted in higher disease severity (percent of spikelets killed).

Using non-parametric correlation analysis and stepwise logistic regression analysis of environmental data collected at 50 location-years in four states representing three wheat production regions in the U.S., De Wolf *et al.* [35] identified combinations of weather variables and their occurrence and duration relative to anthesis that accurately predicted FHB outbreaks in the field. They found the most useful predictor variables to be the duration (hours) of rainfall 7 days before anthesis, the duration (hours) when temperature was between 15 and 30 °C 7 days before anthesis, and the duration (hours) that temperature was between 15 and 30 °C and RH was equal to or greater than 90%. In Belgium, Chandelier *et al.* [36] analyzed climatic data in relation to DON accumulation in winter wheat over a 7-year period. During a 24-day period centered on the mean flowering date, they found a strong positive correlation between the number of days with a mean RH above 80% and mean annual DON content in grain.

Kriss *et al.* [37] analyzed the relationships between environmental factors and biological variables (e.g., FHB intensity, fungal biomass, and DON) associated with FHB in wheat using measurements obtained from 150 location-years originating in three European countries. They determined that in the period from 18 days before anthesis to harvest, moisture-related variables such as average RH and hours of RH above 80% had the highest positive correlations with biological variables. They found significant semi-partial correlations between RH variables and DON in harvested grain. A similar study in the U.S. [38] but with longer window lengths (time of planting to crop maturity) showed that moisture- or wetness-related variables such as daily average RH and total daily precipitation were positively correlated with FHB intensity. 

Cowger *et al.* [39] investigated the effects of post-anthesis moisture on DON accumulation in soft red winter wheat under field conditions. Cultivars moderately resistant or susceptible to FHB were inoculated with conidia of *F. graminearum* at mid anthesis (50% of spikes with extruded anthers) and then subjected to different durations of mist ranging from 0 to 30 days. Misting for 10 and 20 days significantly increased DON compared to no misting in both moderately resistant and susceptible cultivars.

**Figure 6:**
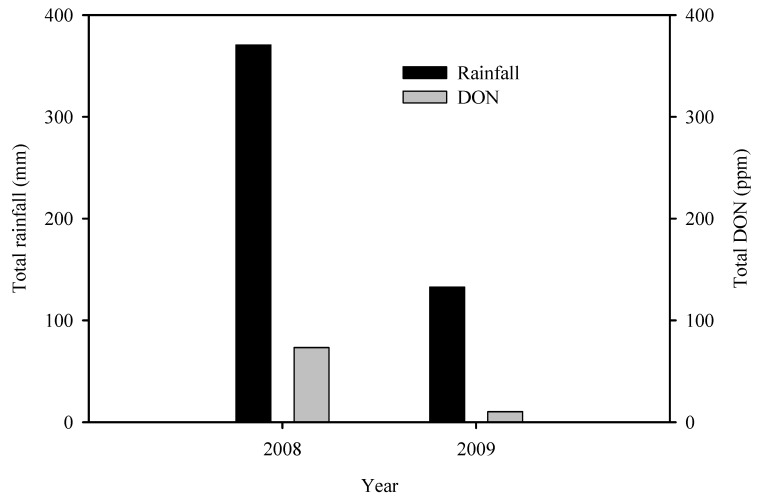
Total rainfall for the months of May and June and total DON in harvested grain in an integrated management field experiment conducted in Nebrasaka, USA in 2008 and repeated in 2009 [30].

Hernandez Nopsa *et al.* [32] observed that in a year in which there was continuous rainfall starting from before anthesis and continuing through grain maturation, DON accumulation in two winter wheat cultivars was much higher than in a year when there was rainfall beginning from before anthesis and continuing through anthesis followed by a dry period after anthesis. Similarly, there was less DON accumulation in the two cultivars in a year in which it was dry prior to and during anthesis followed by rainfall after anthesis. The author compared total rainfall in the months of May and June (the period of highest risk for FHB infections and DON accumulation in wheat in southeastern Nebraska, USA) and total DON in an FHB integrated management study conducted in 2008 and repeated in 2009 [30]. Results showed that total DON was much higher in 2008 when it was excessively wet compared to 2009 when it was relatively dry in the 2-month period (Figure 6). 

The results from these studies indicate that moisture plays an important role in the accumulation of DON in small grain cereals. However, free moisture can also leach out DON from *F. graminearum*-infected wheat spikes. In field experiments, Culler *et al.* [40] showed that DON levels were lower in wheat grain from plots that were subjected to extended irrigation (from anthesis to harvest) compared to grain from plots that were irrigated from anthesis to the early dough stage. In greenhouse experiments, Gautam and Dill-Macky [41] showed that regardless of cultivar, DON levels were lower in winter wheat plants whose spikes were subjected to a single wetting event lasting 6 hours compared to plants that were not wetted. They also detected DON in runoff water from the wetted plants, confirming that DON can leach out from wheat spikes exposed to water such as that from irrigation or rainfall. 

### 4.2. Growth Stage

FHB in small grain cereals develops from infections that occur during the heading growth stages. In wheat, the most damaging infections occur during flowering or shortly after flowering. However, infections can continue as grain maturation progresses. Miller and Young [42] inoculated spikes of winter wheat with spores of *F. graminearum* in field plots followed by misting at 4 and 16 hours, then measured DON concentration in spikes (grain and chaff) over time for up to 9 weeks after inoculation (w.a.i). They found that DON concentration increased up to 9.5 ppm 6 w.a.i then declined thereafter to 2.5 ppm by 9 w.a.i. They attributed the decline in DON concentration after Week 6 to its breakdown by plant enzymes and suggested, based on these observations, that the timing of harvest may influence the amount of DON in grain.

Infections that occur late in grain development can lead to accumulation of DON in apparently healthy grain. In field experiments in North Carolina, USA, Cowger and Arrellano [43] inoculated spikes of eight soft red winter wheat cultivars with spores of *F. graminearum* at 0, 10, or 20 days after anthesis (daa) and then misted the plots for 0, 10, 20, or 30 days starting at anthesis. FDK and DON were found to be higher in the 0-and 10-daa inoculations and in the 0- to 20-daa misted treatments than in later inoculation and misting treatments. However, DON still accumulated to unacceptable levels in the late inoculation treatments. The investigators found that high percentages of low FDK (symptomless kernels) with high DON concentrations occurred under conditions of low disease intensity involving late infection and concluded that late infection is an important factor that results in low FDK and high DON in grain.

In Japan, Yoshida and Nakajima [44] similarly found from greenhouse experiments that *F. graminearum* infections that occurred as late as 20 daa resulted in DON and NIV contamination of grain without visible FHB symptoms on wheat spikes. In a greenhouse study, Del Ponte *et al.* [45] inoculated spikes of spring wheat cultivar Norm with a DON-producing isolate of *F. graminearum* at each of six growth stages from flowering to hard dough. They found that high incidences of kernel infection and significant concentrations of DON resulted from inoculations as late as the hard dough stage, with no corresponding reductions in grain weight. They concluded that FHB-favorable environments well past the flowering growth stage could lead to late infections of wheat spikes and kernels by *F. graminearum*. 

In greenhouse studies, Andersen [34] inoculated spring wheat with conidia of *F. graminearum* at boot (spike still enclosed in the leaf sheath), before flowering (spikes emerged but no anthers extruded), flowering (flowers in anthesis or anthers already extruded), after flowering (small immature kernels present), and past flowering (kernels nearly filled and in the milk to soft dough stage). Plants were then subjected to continuous wetness for 48 hours at 25 °C. Results showed that three days after inoculation, no infection of spikelets occurred in plants inoculated at the boot stage and only 6% of spikelets were infected in plants inoculated at the before flowering stage. However, 79, 98, and 100% of spikelets were infected in plants inoculated at the flowering, after flowering, and past flowering stages, respectively. Five days after inoculation, there was no death of spikelets in plants inoculated at the boot stage and only 4% of spikelets in plants inoculated after spike emergence but before flowering were killed. In contrast, 84, 87, and 100% of spikelets were killed in plants inoculated at the flowering, after flowering, and past flowering stages, respectively.

Susceptibility of the wheat spike to infection by *F. graminearum* at flowering has been attributed to the anthers, which are considered the main site of initial or primary infections [46]. Strange and Smith [27] showed that extruded anthers promoted invasion of the spike by *F. graminerum*. They further demonstrated that extracts of anthers stimulated growth of *F. graminearum in vitro* more than extracts of any other wheat organ, and infection occurred on non-emasculated but not emasculated plants when spikes were inoculated with ascospores of the pathogen. Greater infection of wheat spikes resulted when drops of inoculum were placed on anthers rather than glumes within seven days of anthesis and inoculation of spikes after pollen shed (six weeks after anthesis) did not result in fungal growth or infection of spikelets and spread within the rachis [27]. Stimulants of *F. graminearum* found in wheat anthers were identified as the quaternary ammonium compounds betaine and choline [47,48]. Several studies have shown that susceptibility of the wheat spike to infection by *F. graminearum* declines sharply after the soft dough stage [34,49,50]. Therefore, under field conditions more DON is likely to result from infections that occur from the beginning of flowering to the soft dough stage compared to those that occur at other growth stages.

### 4.3. Inoculum Dosage

In greenhouse experiments, Andersen [34] demonstrated that FHB severity (number of spikelets killed) on *F. graminearum*-inoculated wheat spikes increased with increasing spore concentration, reaching 100% severity at a concentration of two million spores per pot of 10 plants. This implies that in a susceptible cultivar, the higher the concentration of *F. graminearum* spores, the higher the severity of FHB and, therefore, the greater the amount of DON that accumulates in grain. In greenhouse experiments, Stein *et al.* [51] inoculated spikes of a hard red spring wheat cultivar susceptible to FHB with conidia of a highly aggressive isolate of *F. graminearum*. Inoculation was done at the anthesis growth stage and conidial concentration ranged from 0 to 100,000 conidia per ml. Fifteen days after inoculation, FHB incidence (percentage of diseased spikes and severity (percentage of diseased spikelets) were determined and spikes were oven-dried. DON analysis was then done on grain only and whole spikes (grain and chaff). Results showed that FHB incidence and severity increased exponentially and DON concentration in grain only and in whole spikes increased linearly with inoculum concentration. Disease severity did not increase further above a spore concentration of 5,000 spores per spike. However, the level of disease severity reached at this spore concentration differed among replicate experiments. On the other hand, DON concentration continued to increase linearly up to the maximum spore concentration of 25,000 spores per spike reported in the study.

Results from these studies imply that in growing seasons during which environmental conditions (moderate to warm temperatures and prolonged rainfall) favor ascospore production on crop residues, more severe FHB will develop leading to higher accumulation of DON in grain compared to seasons with unfavorable (dry) conditions for disease development. Locally, inoculum concentrations will be higher in fields in which crop residue (maize and wheat stubble) is retained on the soil surface, which is common in reduced or no-tillage systems [23,52,53]. Under these conditions, unacceptably high levels of DON can be expected in grain at the end of the cropping season.

### 4.4. Aggressiveness and Chemotype of Pathogen Isolate

Aggressiveness or virulence is the relative ability of a pathogen to colonize and cause damage to plants [54]. Isolates of a given pathogen can vary in aggressiveness on the same host species. Desjardins *et al.* [55] showed that a trichothecene-nonproducing mutant of *F. graminearum* obtained by disrupting *Tri5*, the gene ecoding trichodiene synthase, was less virulent than a wild-type, trichothecene-producing strain. They inoculated individual florets on wheat spikes in the field at mid anthesis and measured FHB incidence and severity, seeds per spike, total yield per spike, and individual seed weight. Inoculation with the trichothecene-nonproducing mutant resulted in significantly lower disease incidence and severity, more seeds per spike, greater total yield per spike, and greater individual seed weight compared to the wild-type strain.

In Canada, Langevin *et al.* [56] point-inoculated the spikes of six small grain species (bread wheat, durum wheat, triticale, rye, barley, and oats) with two isogenic strains of *F. graminearum* that consisted of a trichothecene- and a non-trichothecene-producing strain. They found that the trichothecene-producing strain was generally more aggressive than the non-producing strain, but the aggressiveness varied with crop species.

In North Dakota, USA, Puri and Zhong [57] investigated the chemotype and aggressiveness on spring wheat of old (collected from 1980 to 2000) and new (collected in 2008) isolates of *F. graminearum*. They found a 15-fold increase in 3-ADON isolates in the new compared to the old collection. Under greenhouse conditions, single-floret inoculation of spikes showed that in a susceptible and a moderately resistant cultivar, the 3-ADON isolates were more aggressive (higher FHB severity) and produced more DON in grain than the 15-ADON isolates. In addition, the 3-ADON isolates produced more DON in rice culture and sporulated more on agar media compared to the 15-ADON isolates.

In Germany, Gang *et al.* [58] inoculated, under field conditions, a susceptible population of winter rye with 42 isolates of *F. culmorum* collected from nine European countries and Australia. They also incubated the same 42 isolates *in vitro* on rye grain. In both assays, the isolates were found to widely differ in the amounts of DON they produced. In the field assay, more DON was produced in grain by the more aggressive isolates, a result similar to that obtained from the *F. graminearum* study by Puri and Zhong [57]. 

These studies show that the more aggressive isolates of *F. graminearum* and *F. culmorum* produce more DON than the less aggressive isolates, and that the acetylated DON chemotype of a given isolate can influence the level of aggressiveness. Under natural field conditions, it is expected that there will be a mixture of isolates varying widely in aggressiveness. However, based on previous studies [13,59] that have shown a correlation between geographical origin and isolate chemotype, a given geographic location is likely to have isolates that are predominantly of one chemotype (3-ADON or 15-ADON). It should be noted that under natural field conditions, environmental conditions (especially moisture amount and duration) may play a more important role in determining the amount of DON produced than the chemotype of pathogen isolates present in a given location. 

### 4.5. Lodging

In the field, lodging is caused mainly by wind, but other factors such as rain, soil type, and nutrient levels (especially nitrogen) in the soil can play a role. Few studies have been done to investigate the effect of lodging on DON accumulation in small grain cereals. Langseth and Stabbetorp [60] conducted field experiments to determine the effect of lodging and time of harvest on DON contamination in barley and oats. They collected grain samples from 19 fields planted separately with a barley or an oat cultivar. Two cultivars of each crop species were included in the study. Half of each field was artificially lodged with a drum roller about three weeks after spike emergence. Grain was harvested at the mealy ripe growth stage and at 15 and 30 days after the mealy ripe growth stage and analyzed for DON content. Results showed that overall mean DON content in grain from the lodged sections of fields (1369 ppb) was nearly twice that from the non-lodged sections (745 ppb). Overall, DON content in grain declined with delayed harvesting.

Nakajima *et al.* [61] investigated the effects of lodging on DON and NIV accumulation in wheat, barley, and rice naturally or artificially infected with *F. graminearum*. They analyzed a total of 66 grain sample sets from fields in which the crops were naturally infected. Each sample set consisted of grain from lodged and non-lodged plants. They also analyzed grain samples from wheat fields in which part of an inoculated area of the field was completely lodged by trampling. In both cases (natural infection and lodging and artificial inoculation and lodging) they found the concentrations of DON and NIV to be significantly higher in grain from lodged compared to non-lodged plants.

These studies indicate that lodging can significantly increase DON concentration in small grain cereals. This increase in DON due to lodging can be attributed to a moist and more humid environment near the soil surface compared to higher locations in the plant canopy where air circulation limits the duration of free moisture and reduces RH. Moisture, RH, and temperature are the three most important environmental factors influencing the growth of *F. graminearum* and the development of FHB (see the *Enviromental Factors* subsection above). Within individual wheat fields, the author has observed more severe FHB in low-lying areas with poor drainage compared to higher ground with good drainage. In addition to moisture and humidity, spikes on lodged plants are in closer proximity to pathogen inoculum on the soil surface, especially in no-till fields that have abundant crop residue. This can result in an increase in infections per spike and therefore an elevated concentration of DON in grain. 

### 4.6. Tillage System and Crop Sequence

Tillage system and crop sequence can significantly influence FHB development and therefore DON accumulation in small grain cereals. Over the last several decades, emphasis has been placed on conservation tillage systems that leave crop residues on the soil surface. These systems conserve soil and water resources and thus contribute to sustainable crop production to meet the food and fiber needs of a growing world population [62]. However, surface residues can provide a suitable habitat for the survival, growth, and multiplication of plant pathogens [63]. Pereyra and Dill-Macky [64] examined the presence of *Fusarium* spp. in residues of various crops common in wheat and barley cropping systems. The residues were collected from no-tillage and reduced-tillage field plots in Uruguay and included residues of wheat, barley, maize, sunflower, pasture, and gramineous weed species. *F. graminearum* was recovered from most of the residues, but most frequently from wheat and barley residues. *F. graminearum* colonized wheat and barley residues longer in no-till than in reduced tillage production systems, suggesting that these residues may be major contributors to FHB inoculum.

Maize and wheat residues are particularly suitable for survival of *F. graminearum*. In Canada, Khonga and Sutton [65] infested maize ears and stems and wheat stems, spikes, and grain with *F. graminearum* and then placed them above, on, or below the soil surface. They observed perithecia formation and spore production on the residues placed on or above the soil surface for up to three years. In Minnesota, USA, Dill-Macky and Jones [66] investigated the effects of previous crop residues and tillage practices on FHB in spring wheat. They determined that FHB intensity was higher when wheat followed maize and lower when wheat followed soybean. They also found that moldboard plowing resulted in lower FHB intensity compared to no-till or chisel plowing. DON concentration in harvested grain was highest when wheat followed maize and lowest when wheat followed soybean. In Italy, Maiorano *et al.* [67] quantified maize residues present on the soil surface and in the top 10 cm of soil in tilled and no-till fields. They then evaluated the influence of the residues on *Fusarium* infection of winter wheat and DON contamination of grain. They found a strong correlation between the total amount of residues and DON contamination of grain. DON levels were higher in no-till fields and in residues on the soil surface compared to tilled fields and buried residues. The investigators concluded that residues on the soil surface played a major role in *Fusarium* infection of wheat and DON contamination of grain.

In Ohio, USA, Selby and Manns [68] found that FHB was more severe in areas where wheat was planted continuously. Hoffer *et al.* [69] and Adams [70] noted that observations in Indiana, Pennsylvania, and Wisconsin, USA showed a greater prevalence of FHB in fields where wheat followed maize in the crop sequence. These observations and the studies mentioned above demonstrate that tillage systems and crop sequence can play a major role in DON accumulation in small grain cereals. Locally, DON concentrations in harvested grain will generally be higher in no-till or reduced tillage systems compared to clean tillage systems and in crop sequences in which FHB-susceptible small grains follow maize or are planted continuously. Recent research by Bergstrom *et al.* [71], however, suggests that even though crop residue (specifically maize residue) can play an important role in DON accumulation in grain locally, regional atmospheric inoculum is the strongest contributor to FHB infections and, therefore, DON accumulation.

### 4.7. Cultivar Resistance

Resistance to FHB in small grains is a complex, quantitative trait [72]. Five categories of resistance have been described [50,72,73,74,75]. They are resistance to initial infection (Type I), resistance to pathogen spread in infected tissue (Type II), resistance to kernel infection (Type III), tolerance (Type IV), and resistance to toxins (Type V). Both native and exotic sources of resistance have been identified in the FHB-prone regions of the world [72,76,77,78,79,80]. Resistance in the Chinese wheat line Sumai 3 and its derivatives is used extensively in breeding programs worldwide [72,77,81]. Many studies have shown that cultivars or genotypes of small grain cereals differ in their resistance to FHB and DON accumulation [29,30,32,82,83,84,85,86]. The majority of these studies have shown that in general, FHB-susceptible cultivars (based on phenotype) accumulate more DON than susceptible ones. However, some studies [32,87] have shown that phenotypically moderately resistant cultivars are susceptible to DON accumulation, suggesting that in some cultivars, resistance to FHB may be independent of resistance to DON accumulation.

The mechanisms by which resistance to FHB and DON accumulation is expressed in small grain cereals are complex and have not been fully elucidated [72]. Miller *et al.* [88] infected nine, five, and six cultivars of spring wheat, rye, and triticale, respectively, with *F. graminearum* and compared fungal biomass (determined as ergosterol) and DON concentration in kernels and chaff. They found that FHB-susceptible cultivars contained higher concentrations of DON in kernels than resistant cultivars and the concentration of DON in chaff in both susceptible and resistant cultivars was approximately eight times that in the kernels. Susceptible cultivars contained higher ergosterol concentrations, suggesting that these cultivars were more susceptible to hyphal invasion. Ergosterol:DON ratios were much higher in resistant compared to susceptible cultivars, implying that resistant cultivars had mechanisms that prevented synthesis or promoted degradation of DON. In a similar study in which 22 wheat genotypes differing in resistance to FHB were infected with *F. graminearum*, Snijders and Krechting [89] also found ergosterol and DON concentrations in the chaff and kernels to be higher in susceptible than in resistant cultivars and concluded that susceptible cultivars were more susceptible to hyphal invasion. They observed that DON was translocated from the chaff to young kernels followed by fungal colonization and that in resistant cultivars, this translocation was inhibited, leading to little fungal colonization in kernels. They attributed the limited fungal colonization of kernels in resistant cultivars to membrane-based trichothecene tolerance.

Sneller *et al.* [29] assessed the genetic variation for resistance to kernel infection and resistance to DON accumulation in 32 wheat genotypes by measuring DON concentration and fungal biomass in grain from spikes with varying degrees of visually estimated FHB intensity. They found some genotypes to consistently have low fungal biomass in grain despite increasing FHB intensity, implying that there was resistance to kernel infection in these cultivars. Some genotypes consistently had low DON concentrations in grain despite increasing FHB intensity, suggesting resistance to DON accumulation. There was lack of significant correlations between resistance to kernel infection and FHB intensity and between resistance to DON accumulation and FHB intensity leading the authors to conclude that these resistances may be independent of Type I and Type II resistances which determine the degree of FHB intensity. 

The results shown in Figure 4 suggest that small grain cultivars susceptible to *Fusarium* kernel damage are likely to accumulate more DON than cultivars resistant to kernel damage. Existence of resistance to kernel damage has been demonstrated in wheat. Bonin and Kolb [90] identified three kernel damage quantitative trait loci (QTLs) in chromosomes 2B, 4B, and 6B in a winter wheat recombinant inbred line population. The 4B QTL explained seven and 12.3% of the phenotypic variation for kernel damage in greenhouse and field experiments, respectively. Because the 4B QTL had previously been identified for FDK [91], the authors concluded that this QTL may contribute to the reduction of FDK in wheat.

### 4.8. Fungicide Application

Several studies have demonstrated the efficacy of fungicides in reducing FHB and DON in small grain cereals under field conditions [30,83,85,92,93,94,95]. Of the two most commonly used fungicide classes (triazoles and strobilurins) for control of diseases in small grain cereals, the triazoles are more effective in controlling FHB and DON than the strobilurins, in part because strobilurins have been shown to be associated with elevated levels of DON in grain. In Italy, Blandino *et al.* [92] found that in field trials, mixtures of triazoles and strobilurins effectively controlled FHB in winter wheat but increased DON in grain (compared to the non-treated check) when conditions were wet. In Hungary, Mesterházy *et al.* [83] observed an increase in DON levels in grain over the non-treated check when azoxystrobin and carbendazim were applied to winter wheat to control FHB in field plots. In China, Zhang *et al.* [95] similarly found increased DON concentrations in grain when azoxystrobin and carbendazim were applied to winter wheat to control FHB under field conditions. 

A multivariate meta-analysis of over 100 FHB uniform fungicide studies across 11 years and 14 states in the U.S. showed that among the triazoles, prothioconazole + tebuconazole (Prosaro) and metconazole (Caramba) were the most effective in controlling FHB and DON [94]. The two fungicides reduced FHB intensity and DON by up to 52 and 45%, respectively. For maximum efficacy in reducing FHB and DON, optimum timing (at anthesis) of fungicide application, using fungicide rates recommended on the label, and thorough coverage of spikes are essential.

Research has shown that combining fungicide application and cultivar resistance is more effective in reducing FHB and DON than using either strategy alone. Wegulo *et al.* [30] showed that fungicide efficacy in reducing FHB and DON was higher in moderately resistant compared to susceptible cultivars. Willyerd *et al.* [85] used data from over 40 trials and 12 states in the U.S. to evaluate the integration of host resistance and application of the fungicide prothioconazole + tebuconazole to manage FHB and DON in small grain cereals. They found that relative to susceptible cultivars not treated with fungicide, the highest mean percent control of FHB (76%) and DON (71%) occurred in moderately resistant cultivars treated with fungicide whereas the lowest mean percent control (43% for FHB and 30% for DON) occurred in moderately susceptible cultivars not treated with fungicide. 

## 5. Regulatory/Advisory Standards for DON

Due to the harmful effects of mycotoxins in humans and animals, many countries have established guidelines or regulatory limits for the maximum levels of mycotoxins in food and feed. In 2003, the Food and Agriculture Organization (FAO) compiled these guidelines and limits in various countries around the world [96]. In the U.S., the Food and Drug Administration (FDA) has established advisory levels for DON as follows [97]: one part per million in finished wheat products for human consumption; 10 ppm in grain and grain byproducts and 30 ppm in distillers grains and brewers grains destined for ruminating beef and feedlot cattle older than 4 months (total ration not to exceed 10 ppm DON), ruminating dairy cattle older than 4 months (total ration not to exceed 5 ppm DON), and chickens (DON-contaminated feed not to exceed 50% of the diet); 5 ppm for grain and grain byproducts destined for swine (DON-contaminated feed not to exceed 20% of the diet); and 5 ppm for grain and grain byproducts for all other animals (DON-contaminated grain not to exceed 40% of the diet). In Canada, maximum DON levels permitted in wheat are 2 ppm in uncleaned soft wheat for use in non-staple foods and 1 ppm in uncleaned soft wheat for use in baby foods [98]. The Canadian Grain Commission has set tolerances for the percentage of *Fusarium*-damaged grain and Agriculture and Agri-Food Canada has established the following DON feeding guidelines: one part per million for swine, dairy cattle, and horses and 5 ppm for poultry and growing beef cattle and sheep [99]. In the European Union, the European Commission has set maximum limits for DON in small grain cereals as 1.25 ppm in unprocessed cereals other than durum wheat and oats; 1.75 ppm in unprocessed durum wheat and oats; 0.75 ppm in cereals for direct human consumption, cereal flour, and bran and germ products marketed for direct human consumption; 0.75 ppm for dry pasta; 0.5 ppm for bread, pastries, biscuits, cereal snacks, and breakfast cereals; and 0.2 ppm for processed cereal-based foods and baby foods for infants and young children [100]. 

## 6. Conclusions

In FHB-prone regions of the world, the disease and the associated mycotoxins, especially DON, continue to pose health risks for humans and animals, as well as economic uncertainty for farmers of small grain cereals and businesses related to grain production and marketing. An understanding of the factors that favor FHB development and DON accumulation in grain is helpful in devising management strategies for the disease and the mycotoxin. These strategies include an integrated approach that combines the use of resistant cultivars, fungicide application at anthesis, FHB and mycotoxin forecasting models, practices that minimize lodging, crop residue management, and a crop rotation sequence that minimizes disease development and mycotoxin accumulation in grain. Additional reduction in DON contamination of harvested grain can be achieved by harvesting severely affected sections of a field separately from the rest of the field, adjusting the combine travel speed and the fan speed and/or the shutter opening of the combine during harvesting to blow out severely damaged kernels which are lighter than healthy kernels [101], using cleaning equipment to remove FDK from harvested grain, and blending *Fusarium*-damaged grain with healthy grain in proportions that will lower DON concentration to levels below regulatory/advisory limits. Regulations that limit the levels of DON and other mycotoxins in food and feed are desirable because they mitigate the health risks in humans and animals associated with the mycotoxins.
